# Genomic and transcriptomic profiling reveals distinct molecular subsets associated with outcomes in mantle cell lymphoma

**DOI:** 10.1172/JCI182153

**Published:** 2024-05-15

**Authors:** Shuhua Yi, Yuting Yan, Meiling Jin, Supriyo Bhattacharya, Yi Wang, Yiming Wu, Lu Yang, Eva Gine, Guillem Clot, Lu Chen, Ying Yu, Dehui Zou, Jun Wang, An T. Phan, Rui Cui, Fei Li, Qi Sun, Qiongli Zhai, Tingyu Wang, Zhen Yu, Lanting Liu, Wei Liu, Rui Lyv, Weiwei Sui, Wenyang Huang, Wenjie Xiong, Huijun Wang, Chengwen Li, Zhijian Xiao, Mu Hao, Jianxiang Wang, Tao Cheng, Silvia Bea, Alex F. Herrera, Alexey Danilov, Elias Campo, Vu N. Ngo, Lugui Qiu, Lili Wang

Original citation: *J Clin Invest*. 2022;132(3):e153283. https://doi.org/10.1172/JCI153283

Citation for this amendment: *J Clin Invest*. 2024;134(10):e182153. https://doi.org/10.1172/JCI182153

Whole-exome sequencing and RNA sequencing data for this study have been deposited in the National Genomics Data Center (https://ngdc.cncb.ac.cn/) under data accession number PRJCA016845.

## Version 1 08/12/2026

Electronic version

